# Telerehabilitation of Developmental Dyslexia: Critical Considerations on Intervention Methods and Their Effectiveness

**DOI:** 10.3390/brainsci14080793

**Published:** 2024-08-07

**Authors:** Claudia Casalini, Chiara Pecini

**Affiliations:** 1 Department of Developmental Neuroscience, IRCCS Fondazione Stella Maris, 56128 Pisa, Italy; claudia.casalini@fsm.unipi.it; 2 Department of Education, Languages, Intercultures, Literatures and Psychology, University of Florence, 50121 Firenze, Italy

**Keywords:** developmental dyslexia, telerehabilitation, efficacy, children, reading

## Abstract

Paper-based or IT tools can be used in telerehabilitation mode to improve the skills of children with developmental dyslexia (DD), seeking to increase reading speed and reduce errors. Telerehabilitation is the provision of remote treatments in which the patient works autonomously in a familiar environment under the remote monitoring, through telecommunication means, of an expert operator. Through telerehabilitation, children with DD can receive treatments outside the specialist clinic, at home or school, via internet connections, and through advanced technological platforms. These procedures allow adequate communication with the family, intensity of treatment, self-adaptivity of exercises, and child engagement; these factors are crucial for a high intervention efficacy. Recent studies have supported the effectiveness of the telerehabilitation of reading in children with DD, with some studies reporting no differences in efficacy between remote and in-person methods. Nevertheless, many points remain to be clarified about the procedures and methods required by telerehabilitation, the variables linked to its effectiveness (e.g., the impact of the intensity of the training and the neuropsychological profile of the child), and the comparative validity of different tele-treatment paths. These aspects are discussed in the present paper.

## 1. Clinical Manifestations of Developmental Dyslexia

Developmental dyslexia (DD) is a specific learning disorder (SLD) primarily characterized by a deficit affecting reading decoding skills (which may be associated with poor reading comprehension and inaccurate spelling skills) occurring in children with normal intellectual abilities and without sensory handicaps or significant neurological and psychopathological disorders who experience an adequate scholastic path and normal educational opportunities [1].

The prevalence estimates of the disorder vary according to the method of diagnosis, the use of a stringent identification cut-off, and the patient’s characteristics (e.g., gender, school level, and writing system) [2,3,4]. However, the incidence, standing globally below 10%, is quite high in the pediatric population (5–15% in children vs. approximately 4% in adults) [5], and DD represents one of the most demanding conditions for the public health system (PHS).

Regarding the causes of DD, studies of families at risk for dyslexia have found the involvement of specific chromosomes (e.g., chromosomes 6, 12, and 15) whose alterations support a polygenic basis of dyslexia [6,7,8,9], although the role of environmental factors that exclude causative genes is also well-acknowledged [10]. Several theories hypothesize that genetic alterations cause functional anomalies in some brain areas and the impairment of various abilities, such as working memory [11], rapid naming [12], language [13], procedural learning [14], processing speed [15], selective attention [16], and executive functions [17]. These processes underpin the automatization of grapheme–phoneme conversion mechanisms and the construction of visual lexicons, which, according to the dual-route model of typical reading, are part of the “indirect” (or “phonological” or “sub-lexical”) and the “direct” (or “orthographic” or “lexical”) route, respectively [18]. The model postulates that, after an initial stage of visual analysis of the stimuli, there are two ways of processing written words: the sub-lexical route involves the identification of letters, the grapheme–phoneme conversion, and finally the articulatory system; the lexical route allows the direct recognition of words without the need to convert each sign in the corresponding sound. An expert reader can use both routes flexibly depending on the type of material to be read and the regularity of the language. In an “opaque” orthography, in fact, the lack of a regular sign–sound correspondence makes the use of the lexical route more necessary and frequent than in a “transparent” orthography, in which, however, it makes reading faster. In children learning to read, instead, the sub-lexical route generally develops first and is more used in the early stages of learning as the sub-lexical route (slower) progressively decreases in favour of the lexical one (faster) [19].

Once sub-lexical and lexical decoding strategies have been acquired, the children can begin to process the content of what they read and develop the ability to understand written text, a complex process that requires the contribution of multiple skills in addition to decoding strategies. According to the model of the “simple view of reading”, for example, reading comprehension depends on both decoding and oral language/listening comprehension abilities [20].

While in acquired adult dyslexia, the two routes can be selectively and differently damaged, in DD they are generally both compromised, although with differences based on developmental stages and the type of language (e.g., “transparent” vs. “opaque”) and for different causes, in many cases also affecting reading comprehension.

Regarding the cognitive underpinnings of DD, the major theories describe DD as a phonological deficit [21], a cerebellar dysfunction [22], a dysfunction of the visuospatial attention system [23], or a disorder of the magnocellular pathways [24].

According to the phonological deficit hypothesis [25,26], DD is a linguistic-based disorder resulting from a congenital dysfunction of the left peri-sylvian brain areas, in which a phonological processing deficit, which interferes with the ability to represent, store, and retrieve speech sounds as well as synthesize them in working memory, hinders the acquisition of grapheme–phoneme correspondence strategies and their use. The temporal processing deficit hypothesis [27] traces phonological problems to an auditory language processing disorder consisting of difficulties in perceiving and processing short or rapidly varying acoustic stimuli, including those crucial for recognizing speech sounds.

The cerebellar (or automatization) dysfunction hypothesis [28] states that alterations of the cerebellum (besides the basal ganglia and motor cortex) lead to difficulties in performing skills automatically and, therefore, cause a procedural memory deficit that hinders the automatization of sign–sound conversion strategies.

Finally, the magnocellular deficit hypothesis [24] claims that a deficit affecting the dorsal magnocellular nervous pathway, responsible for detecting movement and shapes at the periphery of the visual field, translates into difficulties in controlling eye movement and a reduction in the efficiency of the visual attention mechanisms and, therefore, of the visual processing underlying reading [16]. However, since the magnocellular pathway is responsible for the processing of transient signals in other sensory modalities as well, its alterations would generalize to multiple sensory modalities (visual, auditory, and tactile) and to the functioning of the cerebellar system so that a single “cross-modal” biological cause could underly the different functional difficulties of DD.

An alternative hypothesis to that of the magnocellular deficit has recently been put forward. This argues that DD may be associated with a reduction in the facilitation presented by typical readers for stimuli that rely on low-level magno–parvo co-activation compared with stimuli that elicit pure magno activation, i.e., with a perturbation of balance between the magnocellular and parvocellular systems and of their relative contribution in visual processing, with detrimental consequences in word processing, both within the parafovea and the fovea during fixation [29,30].

To date, the evidence in favour of the different theoretical positions and, above all, the heterogeneous functional profiles manifested by DD subjects (who therefore cannot be classified in a single specific model), make it difficult to definitively accredit a theory rather than another [31,32,33]. Therefore, the prevailing view is that, like other neurodevelopmental disorders (ND), DD is a complex multifactorial disorder caused by multiple risk factors, both genetic and environmental, which works according to probabilistic rules and manifests itself at three levels (neurobiological, cognitive and behavioural), in turn interacting with the environment [34]. Specifically, an altered/atypical maturation (determined at a genetic level) of some brain areas and circuits involved in the development of diverse cognitive underpinnings of the reading process, would hinder their functioning, causing the behavioural phenotype of DD to take on various forms, in relation to which skills are altered and to the presence of protective or aggravating environmental factors [35,36,37,38]. Consequently, in addition to cognitive and behavioural assessments, various brain imaging techniques have been applied to look for neuronal markers of dyslexia even at a very early age (e.g., ERPs to speech sounds can differentiate children with and without risk for dyslexia and also show reliable predictive correlations to later language development and reading acquisition) [39,40,41].

This position explains the different clinical manifestations of DD, which show high inter-individual variability in terms of severity and type of decoding difficulties (e.g., slow and/or incorrect reading, inefficient grapheme–phoneme conversion, or visual lexicon construction), association with poor reading comprehension and inaccurate spelling skills, and presence of other subtypes of SLD [42], ND, or other clinical conditions [43].

Furthermore, it should be noted that DD significantly interferes with the individual’s academic (and then professional) achievement and general functioning. Although DD is a primary (i.e., not attributable to other relevant clinical conditions) and specific (i.e., affecting specific aspects of cognitive functioning) disorder, its core deficit, the inadequate mastery of decoding procedures, significantly interferes with other more complex scholastic skills and with study in general. Since transcoding skills require a high cognitive effort, subjects with DD take a long time to complete common school activities and study. They experience a considerable cost in terms of attentional resources and fatigue.

Accordingly, DD can also significantly impact relational experiences and quality of life, especially when it is characterized by comorbidity with other SLD subtypes and the coexistence of neuropsychological difficulties [44] or emotional and behavioural problems [45,46,47,48,49,50]. However, in many cases, emotional–behavioural disorders can be considered a consequence of the emotional distress and social–emotional challenges faced by DD subjects, as a result of their academic difficulties. If left untreated or treated late, DD not only has a negative impact on academic success [51] but is often accompanied by significant social–emotional difficulties [52,53,54] and a decrease in employment opportunities and quality of life in adulthood [55,56,57]. Thus, DD can be seen also as a risk factor for the development of internalizing and externalizing symptoms, impacting mental well-being and increasing the risk of future psychological difficulties and social dysfunctions.

According to the described framework, a timely intervention, both diagnostic and rehabilitative, is mandatory. Appropriate intervention programs must prevent as much as possible short- and long-term negative consequences on the academic curriculum and work opportunities, as well as protect DD subjects’ present and future psychological well-being [58].

## 2. Clinical Management and Treatment Methods in Developmental Dyslexia

Diagnostic and screening processes have improved significantly over the last few years due to a growing understanding of the cognitive deficiencies underlying DD (“cognitive endophenotype”) and the various manifestation profiles (“behavioural phenotype”).

Guidelines for the best operational practices have been developed to identify the deficits in the diverse reading processes and the underlying cognitive skills [59]. Furthermore, screening and prevention have been encouraged to identify subjects at risk of developing DD before exposure to formal learning takes place.

However, the sharing of knowledge on rehabilitation and intervention methods is still limited and, although multiple treatments or intervention programs are available to ameliorate DD symptoms in children [60], the recommended rehabilitation procedures are not well-defined and there is currently a large debate about which therapeutic approaches are more useful and effective.

The treatment of children with DD is, in fact, a complex process and requires intervention at multiple levels, considering various aspects of their functioning and life context (primarily family and school). Different professional figures and different methods can take part in the rehabilitative process in relation to the type, severity, and extent of instrumental and functional deficits, their impact on academic performance, and the associated emotional and social difficulties. After all, although DD is a permanent condition, the children can still recover at least part of their difficulties and can learn strategies that can help them mitigate the impact on school activities and daily life.

In dealing with the reading disorder, three main intervention approaches can be used: (1) “restitution” or “enabling”, a direct approach seeking to “repair” the damaged or poorly developed reading ability or stimulate the development of the reading skill that has not yet emerged; (2) “facilitation”, an indirect approach providing support strategies for the impaired reading ability; and (3) “compensation”, an indirect approach providing replacement strategies capable of alleviating the consequences of the reading disorder.

“Restitution” is the focus of the specialist intervention, which must be aimed at enabling reading skills and therefore encouraging the automatization of basic processes, the defect of which represents the core deficit of DD, especially in the initial stages of schooling. “Facilitation” and “compensation”, on the other hand, constitute initially supportive aspects that can subsequently become priorities in relation to the type and severity of the difficulty and the progress of the schooling. Most importantly in the school context, the use of compensation (i.e., devices and strategies that allow people to detour the limits in reading) and dispensation methods (i.e., measures exempting instrumental errors from penalisation) can promote learning in students with DD, limiting as much as possible the negative consequences of reading difficulties on the study, but also on subject psychological well-being.

Moreover, children with DD can often run into emotional and motivational problems [49,50] such as, for example, reduction in self-esteem and sense of self-efficacy, performance anxiety, and a tendency to abandon the task at the first difficulties; thus, psychological support interventions can play a crucial role in coping with the aforementioned aspects, which can negatively influence the children’s perception of their own abilities and their own value as a student as well as a person, having an unfavourable impact also on the success of the specialist treatment.

More specifically, with regard to the methods of treatment of the typical difficulties of DD, a “direct” intervention on DD consists of working on the “core deficit” of the disorder to reduce the most typical difficulties as much as possible, seeking, for example, to increase reading speed and reduce the number of errors. Various direct methods have been developed, in which the enabling aim can be pursued through work on the competence (the so-called “skill interventions” or “task interventions”, aimed at directly stimulating the compromised reading competence) or on related cognitive abilities (the so-called “process interventions”, aimed at enhancing the cognitive skills that support the development of the impaired reading ability).

The first type of intervention focuses on reading skills and, being aimed at directly strengthening the two routes of reading, can be divided into “sub-lexical” and “lexical” treatments. The sub-lexical treatments facilitate the recognition and processing of sub-lexical parts of words (i.e., letters, syllables, or morphemes) and their association with their phonological correspondences, and consist of exercises on alphabet or syllable identification and grapheme–phoneme conversion rules. Conversely, the lexical treatments enhance direct access to the word by stimulating the ability to recognise the whole word throughout exercises of fast recognition and word mastery (e.g., by a tachistoscopic rapid presentation on the computer screen) without having to break it down into its subunits. Generally, sub-lexical treatments are particularly adequate in the early stages of literacy acquisition, in which the speed of processing letters or syllables is crucial in the word decoding process, while lexical treatments are more adequate in the later stages.

The second type of intervention, instead, belongs to the neuropsychological approach because it tries to enhance the cognitive underpinnings rather than reading itself, and focuses on cognitive processes such as visual–spatial processing, memory, naming, and psychomotor functions. These treatments are useful whenever there is an alteration of a cognitive process involved in reading; they can therefore also be used for preventive purposes in young children who present anomalies in their neuropsychological profile before exposure to learning to read.

In a small number of cases, interventions address both reading skills and the underlying cognitive processes [61] (for a systematic review).

Recent neurophysiological approaches in DD have aimed at stimulating the brain areas that support processes involved in reading in order to increase their efficiency. For example, non-invasive brain stimulation (tDCS) transitorily modulates reading by facilitating the neural pathways that are underactive in individuals with dyslexia [62]. Similarly, the use of color filters (in particular the green filter) improves reading performance in children with DD because the filter most likely facilitates cortical activity and decreases visual distortions [63].

Furthermore, in addition to poor mastery of decoding procedures, children with DD may experience difficulties in reading comprehension and spelling, especially in the advanced stages of schooling and in subjects with atypical linguistic development [13,64]. Thus, direct interventions can also address text comprehension and spelling skills. Since reading comprehension is a multicomponent ability that requires the activation of different cognitive processes to extract from the text, retain and process information, and connect it together into a coherent whole, “process interventions” prevail in the treatment of text comprehension difficulties, aimed at empowering the different skills involved in this task. Some of these skills are basic, such as letter and word recognition, and others complex, such as language processing (in aspects, for example, of grammar and vocabulary knowledge, but also of metaphorical and figurative language), working memory, making inferences, and metacognitive skills such as monitoring the meaning of the text and using strategies such as looking back at the text to resolve ambiguity [65,66,67]. Although reading comprehension disorder can be the result of poor decoding, in many cases, it can occur in the absence of DD. Children who can decode and write correctly but have problems understanding the meaning of what they read are called “poor comprehenders” [68].

Finally, even the spelling difficulties that children with DD may present can be treated both with task interventions, aimed at exercising oral–written transcoding strategies and with process interventions, aimed at stimulating the cognitive processes (e.g., phonological working memory) supporting these skills.

In light of the abovementioned theories, the choice of one of the two approaches takes place based on an accurate clinical evaluation and the most exhaustive definition of the neuropsychological and functional profile of the DD individual. Planning a specific rehabilitation intervention may imply considering various aspects such as the extent and type of reading disorder, the presence of other cognitive deficits or associated problems, the child’s age, educational level, and reading experience, as well as the type of orthography.

In this last regard, comparative studies on the evolution of reading in DD subjects with a “transparent” or “regular” language, such as Italian or German, and with an “opaque” or “irregular” language, such as English [69,70], found that in the former, accuracy is less problematic than speed (“speed dyslexia”) [71], while in the latter, the problem of decoding accuracy remains fundamental, such as that of reading comprehension.

Regardless of the type, to be effective, a treatment must produce a clinical improvement in reading abilities of a magnitude greater than that recorded in the natural evolution of the disorder, perceived as such by the patients or their family and stable over time for a period of at least six months [59,72]. According to the threshold proposed by some authors [73], effective reading interventions on decoding in DD (a more specific subject of this review, focused on the treatment of the main deficit of DD) should overcome improvements seen in untreated individuals over a year, with an increase between or above 0.15–0.30 syllables per second (sill/s) in speed (for decoding of non-words and for decoding of words and texts, respectively) and 50% in accuracy.

The effectiveness of interventions in DD is linked to their timeliness, intensity, and frequency: the lack of early rehabilitation, even before the manifestation of a full-blown and intensive DD, repeated over time, can have long-term effects on educational and professional opportunities and success, causing a high incidence of overlap with other psychological disorders [59]. However, the possibility of early treatment clashes with a series of factors; some are linked to the organization of territorial services (e.g., high costs for the PHS, long waiting lists which often lead to delayed treatments), and others are linked to the characteristics of the subjects and the organization of their family (e.g., inability to access a service).

## 3. Telerehabilitation of Developmental Dyslexia: Theoretical, Practical and Methodological Aspects

The “direct” intervention on the “core deficit” of the DD (both “skill intervention” and “process intervention”) can be carried out, using paper-based or IT tools, both in face-to-face and in telerehabilitation mode [74]. Compared to telerehabilitation, face-to-face intervention has some practical disadvantages. Firstly, it can have limited accessibility to individuals who live in remote areas or face transportation challenges; secondarily, by requiring the co-presence of the child and the clinician, it may imply loss of time, commitment, and a need to change the family ménage, as well as greater expenses for the family itself but also greater costs for the PHS. Furthermore, in relation to the waiting lists and the number of subjects cared for by the clinician, face-to-face care cannot be frequent and intensive, as instead required [59]. In particular, face-to-face therapy occurs at regular intervals (ranging from weekly to monthly) with sessions generally following a structured format with a duration lasting around 45–60 min. All the mentioned characteristics could contribute to a higher response to intervention by telerehabilitation than by face-to-face interventions. In fact, it is acknowledged that in face-to-face treatments, intervention response is a critical point as about 10% of the general student population [75] and up to 50% of students with learning disabilities [76] show an insufficient response to scientifically substantiated reading interventions. However, apart from one study [77] suggesting higher efficiency but equal efficacy of the remote than in-person interventions, to date, no scientific evidence has been provided.

Telerehabilitation consists of the provision of remote rehabilitation treatments in which the patient works autonomously in a familiar environment but under the remote monitoring of the clinician using telecommunication means [78,79]. As a clinical act, telerehabilitation must allow the possibility for patients and operators to interact and for the latter to monitor performance at any time, adapting the parameters of the rehabilitation exercise to the functional profile of the patient and integrating, if necessary, the intervention with other activities [80,81,82].

For DD children, through telerehabilitation interventions, it is possible to receive treatment outside the specialist clinic, at home or school, via internet connections and through the use of advanced IT and technology platforms that allow communication between the clinician and the family. DD subjects involved in telerehabilitation programs are required to follow training sessions for short (lasting approximately 15–20 min) and frequent (multi-day) sessions (at least three days a week), for set periods (on average three months). The mode of the treatment can be synchronous (the clinician leads the activity through a direct connection with the subjects and monitors their activity online), asynchronous (the subjects carry out their activity autonomously according to the clinician’s indications), and mixed (the integration of the two previous ones), and can take place both in a clinical and home setting. This approach shows advantages with respect to face-to-face intervention, on quality of the clinical service provided (facilitate access to care), on the intensity of the rehabilitation service (which can take place in several weekly sessions at home at the most suitable times for family organization), and on reducing costs for the PHS and for families. In particular, the rehabilitation mixed intervention (integrated into the clinic and at home), supervised by the clinician, allows us to guarantee a greater frequency of treatment that can be individualized and therefore built ad hoc by the clinician for every patient and for their specific disorder, and can gradually be modified depending on the trend of their performance (self-adaptivity of the exercises). Furthermore, this type of caring procedure can provide treatment to a greater number of users, thus reducing the waiting lists. Moreover, through telerehabilitation, it is possible to guarantee effective treatment of DD as it is early and timely (not dependent on the long waiting lists of services and available to multiple patients at the same time), intensive (carried out more frequently in the most suitable times for the family), individualized (with activity programmed and monitored based on the characteristics of the children and their disorder and performance trends), motivating both for the child (game-based, so making computer activities pleasant and attractive) and for the family (relieved from the inconvenience caused by a high frequency of visits to the office and sustained by the clinician).

Although already widespread in the rehabilitation of children with SLD for some time, the use of telerehabilitation has increased following the Coronavirus pandemic experience [83], being a modality that has allowed children to be cared for while respecting social distancing imposed to address health issues [84,85,86]. In recent years, various platforms and applications for telerehabilitation of the DD have been developed and there is various evidence in favour of the usefulness of these methods, offering a better solution to the insufficient response to scientifically substantiated reading face-to-face interventions [75,76].

Regarding the method, the use of telerehabilitation for DD requires compliance with precise intervention procedures, the principles to follow and the precautions to take.

1. An accurate multidisciplinary planning phase and logistical support are required for the user for whom computer literacy and the availability of technological devices could affect treatment adherence [87].
2. As for traditional rehabilitation, ethical and legal aspects (e.g., protection of privacy) must be respected for patient safety during and after telerehabilitation sessions [88].
3. Telerehabilitation must not replace but integrate outpatient intervention, to encourage timely, intensive interventions based on therapeutic continuity [89].
4. Self-adaptivity and personalization of the rehabilitation exercise contribute to the effectiveness of tele-treatment. Thanks to the possibility of controlling performance and manipulating exercise parameters remotely, it is possible to customize the proposed activities (e.g., work times, types of exercises, etc.). Self-adaptation, whose parameters must be defined on the basis of precise theoretical models, allows us to personalize the exercise based on the child’s cognitive profile and the change during treatment [88,90], promoting error-free learning, with repercussions also on motivation.
5. The possibility of monitoring the progress of performance online while the treatment is underway, and therefore the progress of the response to treatment, is another important advantage of telerehabilitation methodologies. Some recent studies suggest that the analysis of the learning curve is a more sensitive functional indicator, compared to pre-post differences, of the maintenance of long-term effects and of the generalization of the intervention to other domains (e.g., the improvement curve in a working memory teleintervention in primary school children predicts long-term mathematical skills) [91]. In fact, in telerehabilitation of DD, a high variability of response is observed, with some children who have an initial surge in performance, and therefore can interrupt the intervention earlier than expected, and others who instead progress slowly and systematically could benefit from its extension.
6. Finally, given that the telerehabilitation setting directly involves the parents, who therefore can better understand the difficulties and progress of their child, it is important to constantly discuss parents’ reports and observations, encouraging them to promote the motivation and reinforcement necessary for their child to face the challenges of the exercise.

In light of the criteria described above, the methodology for applying telerehabilitation to DD can be summarized in five main phases:

1. Accurate clinical evaluation of the child’s scholastic learning and cognitive/neuropsychological profiles.
2. Choice (among those based on methodologies or software with scientific evidence) of the most suitable application considering the functional profile and the short-term objectives set.
3. Comparison and alliance with the family. Definition of a collaborative relationship with the family and of a therapeutic contract, making the role of the parent explicit, and highlighting the importance of the systematic nature of the exercise, the duration of the treatment (daily and overall), and constant communication with the clinician.
4. Systematic monitoring of the progress of the treatment and eventual modification of the parameters.
5. Final evaluation to evaluate the changes in the child’s scholastic learning and neuropsychological profile and to redefine new objectives of the intervention. Moreover, considering that telerehabilitation primarily occurs at home, the final evaluation should include social validity. This refers to the acceptability of and satisfaction with the intervention procedures, usually assessed by opinions from the people who receive them and should encompass measures beyond reading skills, such as participant satisfaction, the attitudes of parents and teachers, and community opinions. Some studies are reported in the literature evaluating social validity for families of autistic children who receive telemedicine services or a combination of telemedicine and in-person services [92], while social validity is poorly evaluated for teleintervention on reading skills of DD children, probably also due to the lack of tools to evaluate the perceived quality, satisfaction, and acceptability of telepractice from the perspective of families [93]. For this purpose, Martínez-Rico and colleagues [93], for example, described an instrument aimed at evaluating the social validity of telepractice and usable in early childhood intervention, including a variety of social validity indicators consistent with a family-centered approach such as usability, effectiveness, feasibility, utility, intervention with natural caregivers, and future intentions, sensitive enough to capture differences between the type of service delivery families received.

## 4. Telerehabilitation of Developmental Dyslexia: Feasibility and Effectiveness

Generally, the first question that scientific studies on telerehabilitation try to answer concerns the feasibility and pleasantness perceived by children and their families [94,95,96]. In the field of cognitive telerehabilitation and learning, these, investigated in some studies using questionnaires and structured interviews, are high [97,98,99].

As regards effectiveness, the literature is inconsistent due to the different study designs used, the heterogeneity of the population examined (e.g., linguistic characteristics, age, and school level), the selection criteria of the participants (e.g., type and severity of difficulties), and the tests used to evaluate treatment outcomes. The effectiveness of telerehabilitation can be determined by three crucial factors: the intensity of treatment, the self-adaptivity, and the motivation of both the children and their family.

Telerehabilitation interventions generally support the improvement of the directly exercised function (“near effect”) while a smaller number of studies demonstrate effects on “distant” functions but related to the skill being treated (e.g., improvement in writing following an intervention on decoding reading) [89,100]. A recent systematic review of the literature on the use of cognitive telerehabilitation in neurodevelopmental disabilities highlights that it was found to be more or as effective as the traditional intervention in 47–53% of the studies examined, never proving to be less effective, although the strong and moderate levels of evidence, represent only 36.5% and 24.5% of cases, respectively [101].

Recent studies have demonstrated the effectiveness of software aimed at home rehabilitation of reading disorders in DD children, both through a skill intervention [77,102,103,104,105] and a process intervention, based on exercises stimulating the basic or higher-order cognitive processes that underlie reading [89,106,107,108]. Intervening on transversal cognitive processes, such as attention, working memory and executive functions can simultaneously favour many other learning and adaptive skills, e.g., [109], and it seems to respond to frequent alterations in various other atypical neurodevelopmental conditions.

Several studies documented the effectiveness of telerehabilitation of reading decoding deficits with aloud reading exercises of words or texts of varying difficulty, with a progressive increase in the speed of stimulus presentation [77,83,89,102,104,105]. The reading unit, syllable or word, is usually highlighted and paced according to the child’s reading speed and accuracy at the beginning of the training and at the end of each session. In Cancer’s study [86], the reading exercises, including syllabic blending, syllabic reading, word recognition, and sub-lexical decoding included also simple rhythmic–melodic stimulations coordinated with a visual cue, thus supporting decoding processes by rhythmic and multimodal information. The study by Lorusso and collaborators [99,110] used an intervention program integrating exercises aimed to improve both decoding strategies and visual–spatial attention abilities by tachistoscopically presenting the stimuli in one visual hemifield at a time.

Concerning the interventions focused only on the cognitive underpinning of the reading processes, in the study by Pecini and colleagues [89,100] dyslexic children were trained to improve the naming speed of matrices of black and white figures (RAN), without exposure to alphanumeric stimuli. The results showed comparable efficacy of the RAN training in comparison to similarly structured training in reading skills.

Studies comparing remote treatment with in-presence treatment report no differences in efficacy between the two modalities [86,99,110,111].

Regarding studies on the effectiveness of teleintervention on reading comprehension, most of the research has been conducted in the school context, on typically developing populations and sometimes with technologies that do not allow remote interaction and therefore do not fit into a rigorous definition of telerehabilitation [112]. However, the results of a recent study that used self-adaptive remote intervention show a significant improvement in performance in children with initial weakness in comprehension of the written text [113].

Although not targeting specifically DD, it must be mentioned that remote intervention has also been widely used within the school context to promote decoding skills in children at risk of reading difficulties or poor readers at the beginning of the learning phase. Among several home-based programs, the GraphoGame project is worth mentioning. GraphoGame is a home-based app that responds to all criteria for remote intervention (i.e., self-adaptivity, game-based practice, intense exercises) for empowering children’s early literacy skills, such as grapheme–phoneme correspondences, which has been found effective across different needs and orthographies [114,115,116,117].

A crucial aspect that must be mentioned in order to guarantee the quality of the intervention is intervention fidelity, which is a multidimensional construct that includes adherence (content or steps delivered), quantity (exposure or participant dosage), quality (how well the intervention was delivered, subject responsiveness), and process (program differentiation) [118]. Measuring fidelity is a direct method as opposed to indirect methods (e.g., self-report questionnaire or interview) [119] to interpret findings from interventions [120,121]. In order to directly measure fidelity, an “intervention fidelity reporting” can be used. This measure describes the degree to which an intervention is implemented as planned. This reporting may consist of checklists completed by trained observers who evaluated the implementation of the intervention and the provision of technology [122]. Unfortunately, in most studies on reading treatment, treatment fidelity data are limited; thus, the way in which data on treatment fidelity in intervention studies are collected and reported needs to be improved. Capin and colleagues [123], for example, examined how treatment fidelity is supported, measured, and reported in reading intervention studies conducted with students at risk or with reading difficulties in grades K–3 from 1995 through 2015. Their results indicated that only 47% of the reading intervention studies synthesized reported treatment fidelity data (quantitative or qualitative) and in an incomplete form. Intervention fidelity reporting is scarce also in telerehabilitation studies [124,125] and insufficient evidence exists to draw clear conclusions from it about the efficacy of teleintervention on DD [111].

Psychophysiological research on dyslexia not only helps in the early identification of the disease, e.g., [126,127] but also has opened an interesting window into the study of remediation. fMRI, ERPs and MMN studies, in fact, showed short-term and long-term effects of training on the functional organization of the brain, suggesting the possibility of using this information to guide early intervention (and the behaviourally observable results made comprehensible) [40]. This can be a more effective solution, especially at an early age for children with DD, but the studies comparing the effectiveness of traditional speech therapy techniques and these innovative neurophysiological approaches in DD are scarce.

Although the results of the cited studies suggest a certain degree of effectiveness of telerehabilitation in DD, further research should focus on randomized controlled trial designs, with longer follow-up periods, incorporating a qualitative and quantitative evaluation of processes and the impact on users of the telerehabilitation methodology [128,129]. Finally, there is agreement on the need to have multicomponent telerehabilitation programs, which allow training the multiple processes underlying school learning difficulties [100,130].

## 5. Conclusions and Future Perspectives

In summary, telerehabilitation of DD increasingly represents a valid tool and a continuous boost to the clinical and research potential in this area, both by facilitating the implementation of effective integrated interventions and by encouraging data collection and opening up techniques for innovative analysis and interpretation.

Despite the rapid and widespread diffusion of this methodology and the notable advantages described above, it requires some caution. First of all, it is necessary to encourage participation in specific training courses for clinical operators who use telerehabilitation platforms, in order to assimilate the regulations and general procedures of use and, at the same time, the specific knowledge relating to the program that wants to be used. It is then necessary to monitor and update telerehabilitation programs and/or self-adaptive algorithms based on the responses of children and their families, both by deactivating the self-adaptive methods if necessary and by differentiating clusters of responses to treatment. It is then necessary to verify, with structured interviews, questionnaires, and/or simulations, that the family has secure access to the Internet [131], the technical skills to manage the platform, and is able to guarantee support and functional coaching for the child during carrying out the exercise [83].

It is then important to verify the motivation for use and adherence to therapy since, as with traditional rehabilitation, the interaction between the therapist and the child and the involvement of the parents contribute to the success of telerehabilitation [132]. It is necessary to integrate teleintervention with educational paths in which the family and the school are aware of the purposes and characteristics of the rehabilitation path to make the environment sensitive and responsive.

Finally, the effectiveness of teleintervention must also be evaluated on a single case with standardized tools to verify the effect of the intervention and possibly redefine the objectives, remembering that a pre-post evaluation of the effect is always necessary to demonstrate the potential benefit of telerehabilitation.

Telerehabilitation is therefore a tool widely used today for DD, with potential positive effects on not only reading decoding but also reading comprehension, writing, and calculation skills. However, the hypothesized benefits have only been partially verified with specific studies, e.g., [133], and without intervention fidelity reporting and social validity assessment, and many points still remain to be clarified, such as the variables linked to effectiveness (e.g., the impact of the intensity of the training and the neuropsychological profile of the child) and the comparative validity of the different treatment paths. From a future perspective, therefore, we can hope that telerehabilitation favours the collection of efficacy data and opens up innovative analysis and interpretation techniques, allowing the collection of a large amount of longitudinal and multidimensional data on the individual patient during the interaction with the digital device. This approach, also supported by the emerging of many interactive artificial intelligence tools such as Large Language Models (LLMs) [134] that could enhance telerehabilitation, fundamentally changing the way patients and clinicians access and receive information [135], would allow the construction of specific models that explain differences in effectiveness based on the initial functional assessment and learning trajectories as an opportunity aimed at further personalizing telerehabilitation [136].
